# Therapeutic efficacy of *Schistosoma japonicum* cystatin on sepsis-induced cardiomyopathy in a mouse model

**DOI:** 10.1186/s13071-020-04104-3

**Published:** 2020-05-18

**Authors:** Shifang Gao, Huihui Li, Hong Xie, Shili Wu, Yuan Yuan, Liang Chu, Siying Sun, Huijuan Yang, Lingqin Wu, Yongsheng Bai, Qiao Zhou, Xin Wang, Bin Zhan, Hu Cui, Xiaodi Yang

**Affiliations:** 1 Second Affiliated Hospital of Bengbu Medical College, Bengbu, 233000 China; 2 Anhui Key Laboratory of Infection and Immunity of Bengbu Medical College, Bengbu, 233000 China; 3 Basic Medical College of Bengbu Medical College, Bengbu, 233000 China; 4 First Affiliated Hospital of Bengbu Medical College, Bengbu, 233000 China; 5 National School of Tropical Medicine, Baylor College of Medicine, Houston, TX 77030 USA

**Keywords:** Cystatin, *Schistosoma japonicum*, Myocardial dysfunction, Immunoregulation

## Abstract

**Background:**

Myocardial dysfunction is one of the most common complications of multiple organ failure in septic shock and significantly increases mortality in patients with sepsis. Although many studies having confirmed that helminth-derived proteins have strong immunomodulatory functions and could treat inflammatory diseases, there is no report on the therapeutic effect of *Schistosoma japonicum*-produced cystatin (*Sj-*Cys) on sepsis-induced cardiac dysfunction.

**Methods:**

A model of sepsis-induced myocardial injury was established by cecal ligation and puncture (CLP) in mice. Upon CLP operation, each mouse was intraperitoneally treated with 10 µg of recombinant *Sj*-Cys (r*Sj*-Cys). Twelve hours after CLP, the systolic and diastolic functions of the left ventricular were examined by echocardiography. The levels of myoglobin (Mb), cardiac troponin I (cTnI), N-terminal pro-Brain Natriuretic peptide (NT-proBNP) in sera, and the activity of myeloperoxidase (MPO) in cardiac tissues were examined as biomarkers for heart injury. The heart tissue was collected for checking pathological changes, macrophages and pro-inflammatory cytokine levels. To address the signaling pathway involved in the anti-inflammatory effects of r*Sj*-Cys, myeloid differentiation factor 88 (MyD88) was determined in heart tissue of mice with sepsis and LPS-stimulated H9C2 cardiomyocytes. In addition, the therapeutic effects of r*Sj*-Cys on LPS-induced cardiomyocyte apoptosis were also detected. The levels of M1 biomarker iNOS and M2 biomarker Arg-1 were detected in heart tissue. The pro-inflammatory cytokines TNF-α and IL-6, and regulatory cytokines IL-10 and TGF-β were measured in sera and their mRNA levels in heart tissue of r*Sj*-Cys-treated mice.

**Results:**

After r*Sj*-Cys treatment, the sepsis-induced heart malfunction was largely improved. The inflammation and injury of heart tissue were significantly alleviated, characterized as significantly decreased infiltration of inflammatory cells in cardiac tissues and fiber swelling, reduced levels of Mb, cTnI and NT-proBNP in sera, and MPO activity in heart tissue. The therapeutic efficacy of r*Sj*-Cys is associated with downregulated pro-inflammatory cytokines (TNF-α and IL-6) and upregulated regulatory inflammatory cytokines (IL-10 and TGF-β), possibly through inhibiting the LPS-MyD88 signal pathway.

**Conclusions:**

R*Sj*-Cys significantly reduced sepsis-induced cardiomyopathy and could be considered as a potential therapeutic agent for the prevention and treatment of sepsis associated cardiac dysfunction.
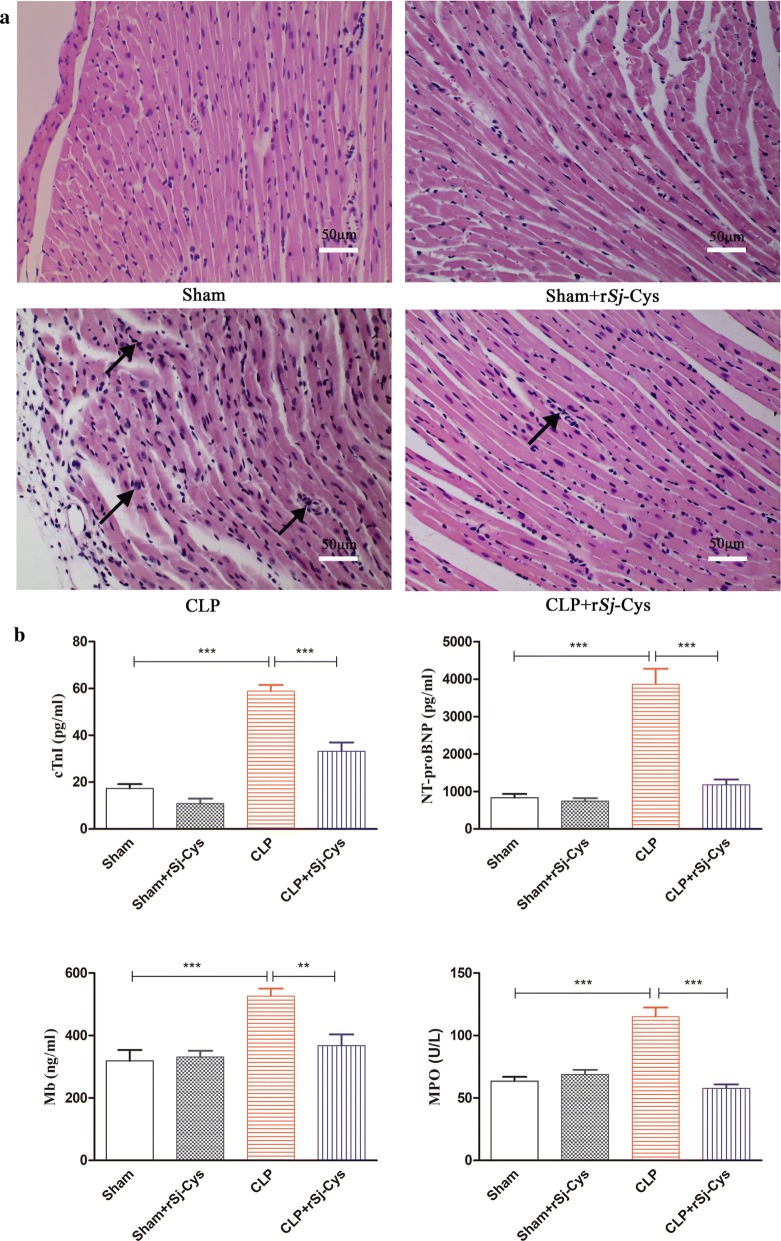

## Background

Sepsis is a life-threatening organ dysfunction caused by serious infection, affecting the lives of millions of people around the world [1, 2]. Myocardial dysfunction is a common complication of hospitalized sepsis patients, and myocardial depression occurs in 40–50% of patients with sepsis [3, 4]. Septic myocardial dysfunction is associated with overproduction of pro-inflammatory cytokines, including IL-6 and TNF-α, which play pivotal roles in cardiomyocyte apoptosis and injury [3, 5]. In recent years, studies have suggested that sepsis-induced cardiac dysfunction, the major cause of sepsis mortality (70–90%) [6], is caused by myocardial apoptosis mediated by the MyD88 signal pathway that activates and over-expresses a variety of pro-inflammatory cytokines, including TNF-α and IL-6 [7]. At present, the control of sepsis-induced cardiac failure depends on drug therapies. The most commonly used drugs for the treatment of sepsis-induced cardiac dysfunction are glucocorticoid, norepinephrine, low molecular weight heparin and antibiotics. Although these drugs are capable of preventing the development of inflammation, activating the anti-coagulation system, enhancing anti-inflammatory function, or suppressing the bacterial proliferation, there is still a proportion of patients who cannot survive from severe sepsis. Other alternative approaches to better control sepsis and reduce sepsis-related myocardial dysfunction is greatly needed.

Parasitic helminths co-evolve with mammalian hosts and develop some strategies to survive within hosts. These strategies include modulating the host immune system to downregulate the immune response to helminths (parasite-specific immunomodulation) [8], characterized by a dominant Th2-mediated immune response and activated regulatory T cells (Tregs) or monocyte responses [9–11]. The helminth-induced regulatory responses not only facilitate the survival of worms in the host, but also benefit the host to reach immune homeostasis between the resistance and tolerance and reduce immunopathology [12]. Helminth infection induced alternately activated macrophages (AAM) [13] and Tregs play important roles in the control of inflammation and tissue repair [13–15]. Further evidence showed that helminth-secreted proteins can induce the host to actively produce immune regulation [16, 17]. Due to their potent immunomodulatory functions, helminth infections or helminth-derived or secreted proteins, have been used as therapeutic regents to treat some immunno-inflammatory diseases such as allergies and autoimmune disorders [18–20]. In particular, cystatins derived from various parasitic helminths have received most of the attention because they have been identified as strong immunomodulatory proteins [14] and successfully used as potential therapeutic agents for inflammatory and autoimmune diseases [21–25]. Parasitic helminth cystatins have been demonstrated to ameliorate arthritis, asthma and colitis [14, 22, 24, 25]. *Sj*-Cys is a cysteine protease inhibitor (cystatin) derived from the blood-feeding trematode *Schistosoma japonicum* [26]. Treatment with r*Sj*-Cys significantly stimulated Tregs and inhibited the antigen-presenting functions of dendritic cells (DCs) [27]. It also inhibited the release of pro-inflammatory factors (TNF-α, IL-6) in LPS-stimulated macrophages [23]. Recombinant *Sj*-Cys has been used as a therapeutic agent to alleviate the severity of dextran sulfate sodium (DSS)-induced colitis in mice [21] and murine collagen-induced arthritis [22]. Our previous study has identified that r*Sj*-Cys displayed the therapeutic effect of cecal ligation and puncture (CLP)-induced bacterial sepsis characterized by the increased survival rates, alleviated overall disease severity and tissue injury of liver, kidney and lungs [12]. These therapeutic effects are associated with downregulation of pro-inflammatory cytokines and upregulation of regulatory cytokines [12].

In this study, we explore the therapeutic effect of r*Sj*-Cys on sepsis-triggered cardiac dysfunction and we found that treatment with r*Sj*-Cys significantly reduced the sepsis-induced cardiomyopathy and heart injury in a mouse model, providing an alternative approach to control sepsis-induced heart failure and death.

## Methods

### Production of recombinant *Sj*-Cys

DNA coding for *Sj*-Cys was cloned in-frame into pET28a and the sequencing confirmed recombinant plasmid DNA with correct insert was transformed into *E. coli* BL21 using the calcium transfection method. The recombinant *Sj*-Cys (r*Sj*-Cys) with a His-tag at the N-terminus was induced with 1 mM isopropylthio-β-galactoside (IPTG; Sigma-Aldrich, Steinheim, Germany) at 37 °C for 5 h, and purified from the soluble fraction of the induced bacteria using a HisPur™ Ni-NTA Spin Column (Thermo Fisher Scientific Inc., Waltham, USA). The contaminated endotoxin was removed from the purified recombinant protein using a ToxOut™ High Capacity Endotoxin Removal Kit (BioVision, Palo Alto, California, USA) and the residual endotoxin level was measured using a ToxinSensor™ Chromogenic Limulus Amebocyte Lysate (LAL) Endotoxin Assay Kit (GenScript Biotechnology, Nanjing, China) following the manufacturer’s protocol. The concentration of r*Sj*-Cys was measured using a Bicinchoninic Acid Protein Assay Kit (Beyotime Biotechnology, Shanghai, China) and the recombinant protein stored at − 80 °C until use.

### Animals

Specific pathogen-free (SPF) 6–8-week-old male BALB/c mice (body weight of 20–22 g) were purchased from Anhui Medical University Experimental Animal Facility (approval no. AMU26-08061). The mice were housed in a climate-controlled facility maintained at 23 ± 1 °C, 55 ± 5% humidity with a 12 h light/dark photocycle and *ad libitum* access to food and water.

### Sepsis-induced cardiomyopathy

The mice were subjected to CLP surgery according to the method described previously [4]. Briefly, mice were fasted for 12 h, with only drinking water available, and then anesthetized by intraperitoneal injection of 0.2 ml/20 g of 4% chloral hydrate. Following a 1–2 cm midline laparotomy incision, 66% of the cecum was ligated with a 4-0 silk suture (Syneture, Norwalk, CT). A through-and-through puncture was made on the anti-mesenteric side with an 18-gauge needle and a small amount of feces was extruded through the puncture holes to ensure perforation. The cecum was placed back in its original location and the abdomen was closed in two layers with 4-0 silk (Syneture). Following CLP, sterile normal saline (300 µl) was injected sub-dermally for fluid resuscitation. Sham mice underwent the above process except for CLP.

### Treatment of sepsis-induced cardiomyopathy with r*Sj*-Cys

A total of 6 CLP-operated mice were treated intraperitoneally with 10 µg of r*Sj*-Cys in a total volume of 200 μl 30 min after surgery. The same number of CLP-operated mice were given 200 µl of PBS only as a control. As normal controls, 12 mice that underwent sham surgery were divided into two groups; 6 received the same amount of r*Sj*-Cys and 6 received PBS only. Twelve hours later, all mice were measured for echocardiography. Blood was collected from each mouse under anesthesia and sera were centrifuged at 3000× *rpm* for 15 min at 4 °C and stored at − 80 °C until use. All mice were euthanized and hearts collected for histopathological staining and measurement.

### Echocardiography

Echocardiographic evaluation was performed using a high-resolution echocardiograph (Vevo 2100; VisualSonics, Toronto, Canada) for the differently treated mice groups. Briefly, a mixture of 1% isoflurane and oxygen was inhaled *via* a nose cone, and each mouse was carefully kept under mild anesthesia and subjected to M-mode and Doppler echocardiography according to the method described previously [28]. The ejection fraction (EF%) and fractional shortening (FS%) of the left ventricle were calculated from M-mode tracing to reflect left systolic function. Peak early-diastolic transmitral velocities (E wave) and peak late-diastolic transmitral velocities (A wave) across the mitral valve inflow were examined on Doppler flow tracings and were used to calculate E/A ratios, a commonly used parameter of left ventricular diastolic function. All echocardiographic procedures were performed by the same skilled operator and data averaged from at least three consecutive cardiac cycles.

### Histological examination of myocardium

Mouse hearts collected from different experimental groups were fixed in 4% buffered paraformaldehyde for 12 h. Fixed left heart ventricles were sectioned and stained with hematoxylin and eosin (H&E) stain. H&E stained sections were observed under light microscopy (200× magnification) (Nikon, Tokyo, Japan) for pathological changes.

### Biochemical analysis

The heart-released myoglobin (Mb), cardiac troponin I (cTnI) and N-terminal pro-Brain Natriuretic peptide (NT-proBNP) in sera, and myeloperoxidase (MPO) in heart tissue, were measured as biochemical markers for heart injury. The levels of cTnI and NT-proBNP in sera were detected using an enzyme-linked immunosorbent assay (ELISA) kit (Elabscience Biotechnology Co., Ltd, Wuhan, China). The concentration of Mb was measured in the mouse sera using a Fully Automated Biochemistry Analyzer (Beckman Coulter, Brea, California, USA). The heart tissue was weighed and homogenized, the MPO activity in the homogenate was determined using a MPO test kit (Bioenginering Institute, Nanjing, China).

### Detection of IL-6, TNF-α, TGF-β and IL-10 in sera and cell supernatants

The concentration of pro-inflammatory (TNF-α and IL-6) and regulatory (IL-10 and TGF-β) cytokines in cell culture supernatants and experimental mouse sera were detected by ELISA in accordance with the manufacturer’s instructions (ABclonal Biotechnology Co., Ltd. Wuhan, China).

### Detection of cardiac TNF-α, IL-6, IL-10, TGF-β, iNOS and Arg-1 mRNA expression by quantitative real time PCR (qRT-PCR)

Total RNA from the left ventricular myocardium was extracted with QIAzol reagent (Ambion, Austin, TX, USA). Then cDNAs were reverse-transcribed from 2 μg total RNA using a reverse transcription kit (RevertAid First Strand cDNA Synthesis Kit; Thermo Fisher Scientific Inc.). The cDNA was used as a template for qRT-PCR using the SYBR Green Super Mix Kit (Takara Bio Inc., Tokyo, Japan). All samples were duplicated and the qRT-PCR signal of the target transcript in the treated group was compared with the control housekeeper gene (GAPDH) signal by relative quantification. The 2^−ΔΔCq^ method was used to analyze the relative change in gene expression. The primers (GAPDH, TNF-α and IL-6) were designed and synthesized by Sangon Biotech (Shanghai, China). The forward and reverse primers of target genes are listed in Additional file 1: Table S1 [29–31].

### Cell culture and treatment

H9C2 rat embryo cardiomyocytes were purchased from the American Type Culture Collection (ATCC, Manassas, VA, USA) and cultured in Dulbecco’ s modified Eagle’ s medium (DMEM) containing 10% fetal bovine serum (Biowest S.A.S, Niayet, France) and 1% penicillin/streptomycin (Gibco, Grand Island, NY) at 37 °C, 5% CO_2_. Cultured H9C2 cells were treated with r*Sj*-Cys (0.5 µg/ml) for 0.5 h, and then exposed to 1 µg/ml of LPS (Solaibao, Beijing, China) for 24 h. Cells incubated with LPS without r*Sj*-Cys treatment, or cells incubated with r*Sj*-Cys or medium alone were used as controls. After 24 h of incubation, the culture was centrifuged at 1000× *rpm* for 15 min at 4 °C, the supernatants were stored at − 80 °C until use, and the cells were used for flow cytometry assays.

### Detection of myocardial cell apoptosis by flow cytometry (FCM)

The LPS-induced myocardial cell apotosis was measured by annexin v-fitc and propidium iodide (pi) staining in accordance with the manufacturer’s instructions (Invitrogen, Thermo Fisher Scientific). Flow cytometric analysis was performed on a CYTEK DxP AthenaTM Analyzer (CyTeK Biosciences, California, USA). The results were analyzed with FlowJo V7.6.5 software.

### Detection of MyD88 by western blotting

MyD88 expression level in treated H9C2 cells and myocardial tissue was determined by western blotting. Briefly, cells or left ventricular myocardium were collected and homogenized in ice-cold RIPA buffer containing 0.1% phenylmethylsulfonyl fluoride. The homogenates were centrifuged at 12,000× *rpm* for 15 min at 4 °C. Supernatants were collected and protein concentration was quantified using a BCA assay kit (Pierce, Rockford, IL, USA). Equal amounts of cell extracts or heart homogenates were separated by 12% SDS-PAGE and electroblotted onto PVDF membranes. After blocking with 5% skimmed milk for 2 h at room temperature, membranes were incubated with rabbit anti-MyD88 antibody (1:800) (Cell Signaling Technology, Danvers, Massachusetts, USA) overnight at 4 °C, followed by HRP-conjugated goat anti-rabbit IgG (1:4000) (Merck Millipore, Basilica, Massachusetts, USA) for 1 h at 37 °C. Immunoreactive protein bands were visualized using a Tanon 5200 Chemiluminescence Imaging System (Tanon, Shanghai, China).

### Statistical analysis

All data are expressed as the mean ± standard error of the mean (SE), and statistical analyses were performed using GraphPad Prism 5.0 software (GraphPad Inc., La Jolla, CA, USA). One-way ANOVA followed by the Student-Newman-Keuls test was used for multigroup comparisons; *P*-value of< 0.05 was considered statistically significant.

## Results

### Treatment with r*Sj-*Cys alleviated sepsis-induced myocardial malfunction

**Fig. 1:**
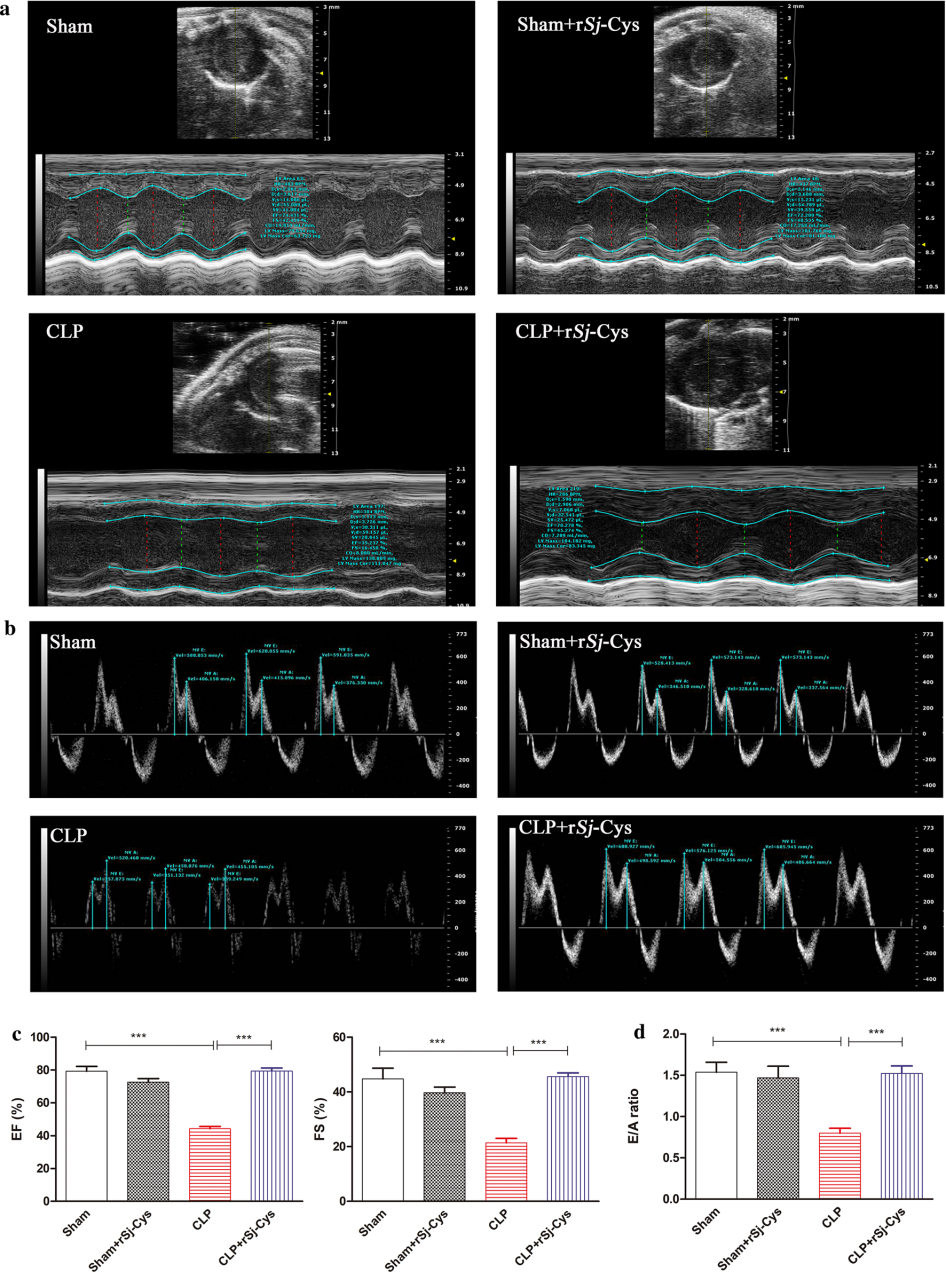
Treatment of r*Sj*-Cys improved the sepsis-induced myocardial malfunction. Representative M-mode echocardiograms obtained from mice 12 h after treatment of sham-operation, sham + r*Sj*-Cys, CLP and CLP + r*Sj*-Cys, respectively (**a**). The improved left ventricular systolic function evaluated by EF and FS after treatment with r*Sj*-Cys (**c**). The improved CLP-induced left ventricular diastolic dysfunction was shown as representative transmitral Doppler images. The E wave represents peak early-diastolic transmitral velocity, and the A wave indicates peak late-diastolic transmitral velocity (**b**). The changes of the E/A ratio were used to assess the alteration in left ventricular diastolic function (**d**). (*n* = 6 mice per group). Data are presented as the mean ± SE. ***P < 0.001

Left ventricular function was examined by echocardiography 12 h after CLP surgery. As shown in Fig. 1a and c, CLP-induced sepsis caused a significant reduction of left ventricular systolic function in mice, characterized by reduced EF and FS compared with the sham operation group (ANOVA: *F*_(3, 23)_ = 63.07, *P *< 0.0001 and *F*_(3, 23)_ = 21.08, *P *< 0.0001, respectively). In contrast, r*Sj-*Cys treatment dramatically reversed the sepsis-induced decrease in the left ventricular EF and FS to a similar level in mice of the sham surgery control group (ANOVA: *F*_(3, 23)_ = 63.07, *P *< 0.0001 and *F*_(3, 23)_ = 21.08, *P *< 0.0001, respectively), indicating that treatment with r*Sj-*Cys reduced the sepsis-induced myocardial systolic malfunction in mice (Fig. 1c). In addition, administration of r*Sj-*Cys in sham surgery mice did not markedly alter left ventricular EF and FS compared with the sham group without treatment (Fig. 1c).

To assess the left ventricular diastolic function, the E/A ratio was calculated from Doppler-derived mitral valve inflow measurements. The results showed that sepsis mice displayed a significant decline in E/A ratio values compared to sham control mice (ANOVA: *F*_(3, 23)_ = 10.99, *P *< 0.0002) (Fig. 1b, d). Treatment with r*Sj-*Cys significantly recovered the E/A ratio to a similar level of mice which had received sham surgery or sham + r*Sj-*Cys (ANOVA: *F*_(3, 23)_ = 10.99, *P *< 0.0002) (Fig. 1b, d). These results indicate that treatment with r*Sj-*Cys also improves sepsis-induced diastolic malfunction in mice. No significant difference was observed between the sham group and the sham r*Sj-*Cys-treated group in terms of E/A ratio (ANOVA: *F*_(3, 23)_ = 10.99, *P *< 0.0002) (Fig. 1d).

### Treatment of r*Sj-*Cys reduced sepsis-induced pathological heart abnormalities

**Fig. 2:**
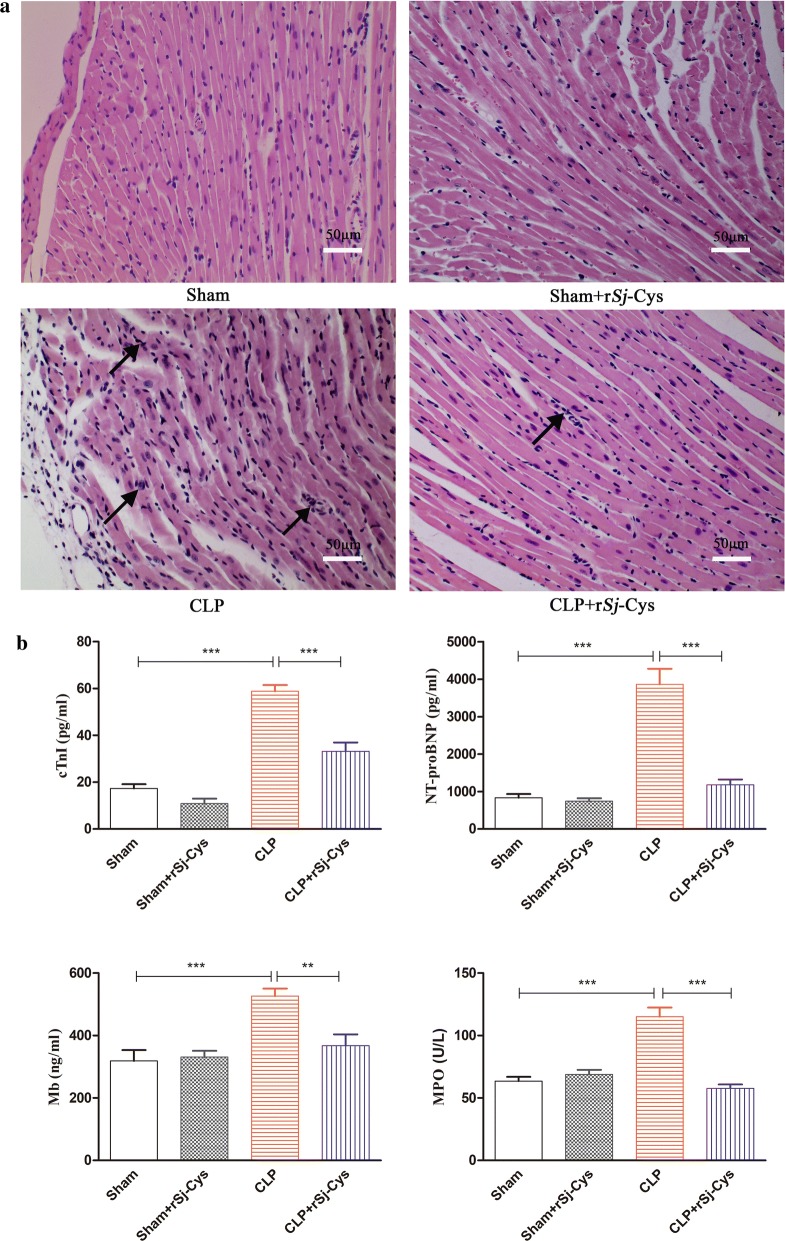
**a** Histopathological and morphological variations in the cardiac tissue of mice following sham surgery, sham + r*Sj*-Cys, CLP and CLP + r*Sj*-Cys (*n* = 6 mice per group, 200× magnification). Treatment with r*Sj*-Cys reduced CLP-sepsis induced myocardial injury in the BALB/c mice. **b** The levels of cTnI, NT-proBNP, Mb in sera and MPO activity in heart tissue were significantly reduced in CLP mice treated with r*Sj*-Cys. The data are presented as the mean ± SE. **P < 0.01, ***P < 0.001. *Scale-bars*: 50 μm

The morphological structures and pathology of the myocardial tissues of mice 12 h after CLP surgery were determined by H&E staining. The results showed that the sham surgery group and sham with r*Sj-*Cys group had no significant inflammatory cell infiltration with normal appearance of the myofibrillar structure (Fig. 2a). However, the heart tissue in mice with CLP showed significant inflammation, myocardial fiber arrangement disorder and highly recruited inflammatory cell infiltration. Of note, tissue sections from the CLP + r*Sj-*Cys mice group showed significantly improved muscle fiber structure with reduced inflammatory cell infiltration compared with CLP group without r*Sj*-Cys treatment (Fig. 2a). The pathological results indicate that r*Sj-*Cys effectively alleviates CLP-induced cardiac lesions and inflammation in mice.

### Administration of r*Sj*-Cys reduced sepsis-induced heart injury

In myocardial injury, released Mb, cTnI, NT-proBNP and MPO into sera or heart tissue are usually used as biomarkers to evaluate the ischemic severity of heart injury [32–34]. Compared with the sham group, the CLP group showed a marked increase in MPO activity in the myocardial tissue homogenate and elevated levels of cTnI, Mb, NT-proBNP in sera (ANOVA: *F*_(3, 23)_ = 32.10, *P *< 0.0001; *F*_(3, 23)_ = 61.36, *P *< 0.0001; *F*_(3, 23)_ = 10.42, *P *< 0.0002; and *F*_(3, 23)_ = 42.79, *P *< 0.0001, respectively) (Fig. 2b). After being treated with r*Sj*-Cys, the MPO activity in the myocardial homogenate and cTnI, Mb, NT-proBNP in sera were significantly reduced in mice with sepsis compared with CLP mice without treatment (ANOVA: *F*_(3, 23)_ =32.10, *P *< 0.0001; *F*_(3, 23)_ = 61.36, *P *< 0.0001; *F*_(3, 23)_ = 10.42, *P *< 0.0002; and *F*_(3, 23)_ = s42.79, *P *< 0.0001, respectively) (Fig. 2b), while cTnI, NT-proBNP, Mb and MPO remained at low levels in mice with sham surgery and there was no significant difference between sham surgery groups and sham with treatment of r*Sj*-Cys groups. The increased MPO activity in the heart tissue of CLP-induced sepsis mice was correlated with the increased inflammatory cell infiltration, especially neutrophils, in the heart tissue (Fig. 2b).

### r*Sj*-Cys inhibits pro-inflammatory cytokines and induces regulatory cytokines in mice with sepsis-caused heart injury

**Fig. 3:**
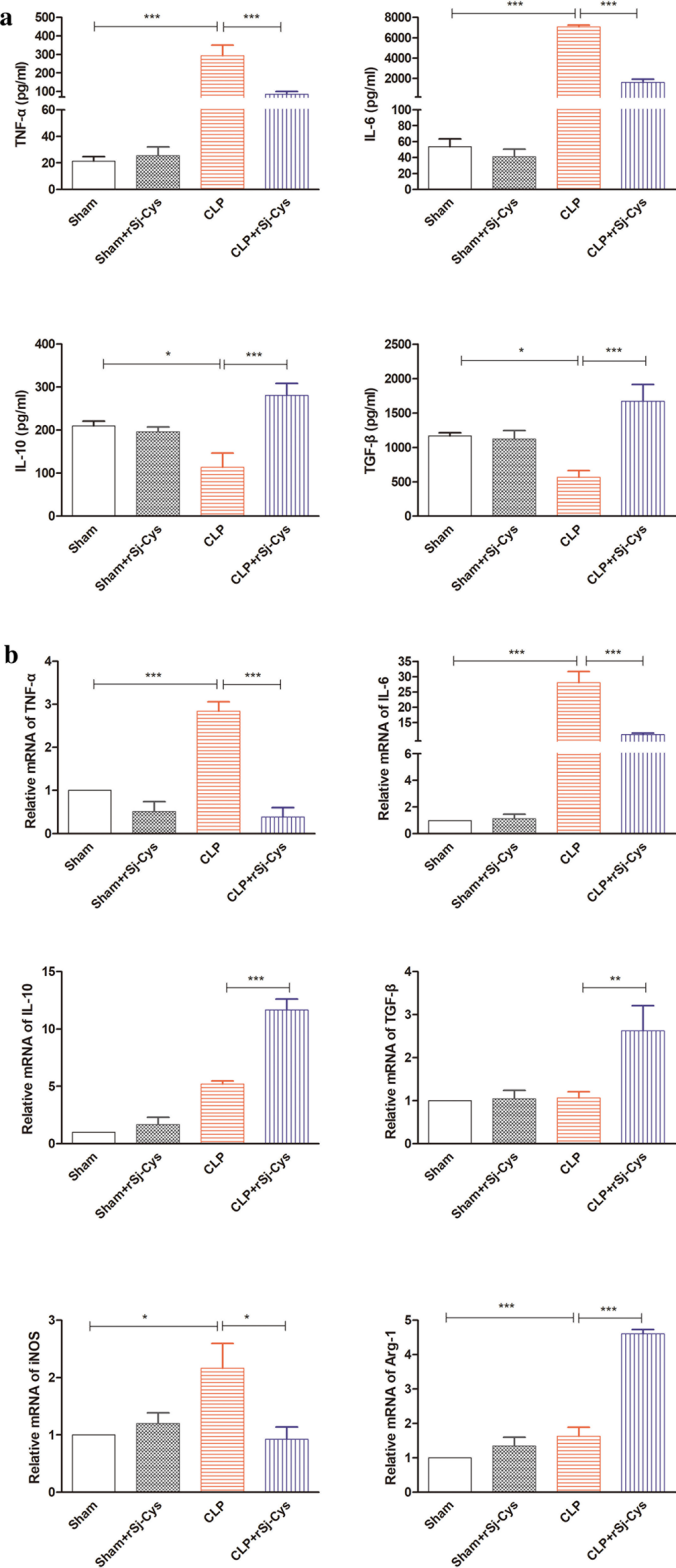
Treatment with r*Sj*-Cys reduced the pro-inflammatory cytokine (TNF-α and IL-6) and boosted regulatory cytokine (IL-10 and TGF-β) levels in sera (**a**) and the similar mRNA expression pattern observed in heart tissues (**b**) of mice with CLP-induced sepsis 12 h after treatment. The mRNA expression level of M1 macrophage marker (iNOS) was reduced and that of M2 macrophage marker (Arg-1) increased in heart tissues (**b**). The data are shown as the mean ± SE for each group (*n* = 3 mice per group). *P < 0.05, **P < 0.01, ***P < 0.001

To understand the mechanisms underlying the improvement of sepsis-caused cardiac dysfunction with treatment of r*Sj*-Cys, the levels of pro-inflammatory cytokines (TNF-α and IL-6) and regulatory cytokines (IL-10 and TGF-β) were measured in sera, and the mRNA levels measured in heart tissue of experimental mice. The results showed that the inflammatory cytokines (TNF-α and IL-6) dramatically increased in the sera of CLP-induced sepsis mice, compared to that in mice with sham surgery only or sham + r*Sj*-Cys (ANOVA: *F*_(3, 23)_ = 18.39, *P *< 0.0001 and *F*_(3, 23)_ = 361.3, *P *< 0.0001, respectively) (Fig. 3a). Treatment with r*Sj-*Cys significantly reduced the production of TNF-α and IL-6 in CLP-induced sepsis mice, compared with CLP mice without treatment (ANOVA: *F*_(3, 23)_ = 18.39, *P *< 0.0001 and *F*_(3, 23)_ = 361.3, *P *< 0.0001, respectively) (Fig. 3a). However, there was no significant difference of TNF-α and IL-6 levels in sera of mice between the sham group and sham + r*Sj-*Cys group (ANOVA: *F*_(3, 23)_ = 18.39, *P *< 0.0001 and *F*_(3, 23)_ = 361.3, *P *< 0.0001, respectively) (Fig. 3a). The reduced TNF-α and IL-6 levels were correlated with the increased IL-10 and TGF-β levels in sera of CLP-induced sepsis mice treated with r*Sj*-Cys compared with the CLP group without treatment (Fig. 3a). The IL-10 and TGF-β levels were significantly lower in CLP-induced sepsis mice than that in sham surgery or sham + r*Sj*-Cys mice (ANOVA: *F*_(3, 23)_ = 9.032, *P *< 0.0006 and *F*_(3, 23)_ = 9.789, *P *< 0.0004, respectively) (Fig. 3a). The mRNA expression levels of pro-inflammatory cytokines (TNF-α and IL-6) and regulatory cytokines (IL-10 and TGF-β) detected in heart tissue showed a similar pattern to that measured in sera (Fig. 3b). The results suggested that CLP-induced sepsis mice stimulated the secretion of pro-inflammatory cytokines (TNF-α and IL-6), but inhibited the regulatory immune pathway (lower levels of IL-10 and TGF-β). Treatment of r*Sj*-Cys was able to significantly inhibit the activation of the pro-inflammatory pathway, possibly by activating the regulatory immune pathway. Interestingly, the mRNA level of the M1 macrophage marker iNOS was significantly reduced, and the M2 macrophage maker Arg-1 significantly increased in heart tissues of r*Sj*-Cys-treated sepsis-mice (ANOVA: *F*_(3, 11)_ = 4.967, *P *< 0.0311 and *F*_(3, 11)_ = 77.27, *P *< 0.0001, respectively) (Fig. 3b), indicating that more macrophages shifted from M1 to M2 after being treated with r*Sj*-Cys.

### The inhibitory effect of r*Sj*-Cys on LPS-induced inflammatory response in H9C2 cardiomyocytes

**Fig. 4:**
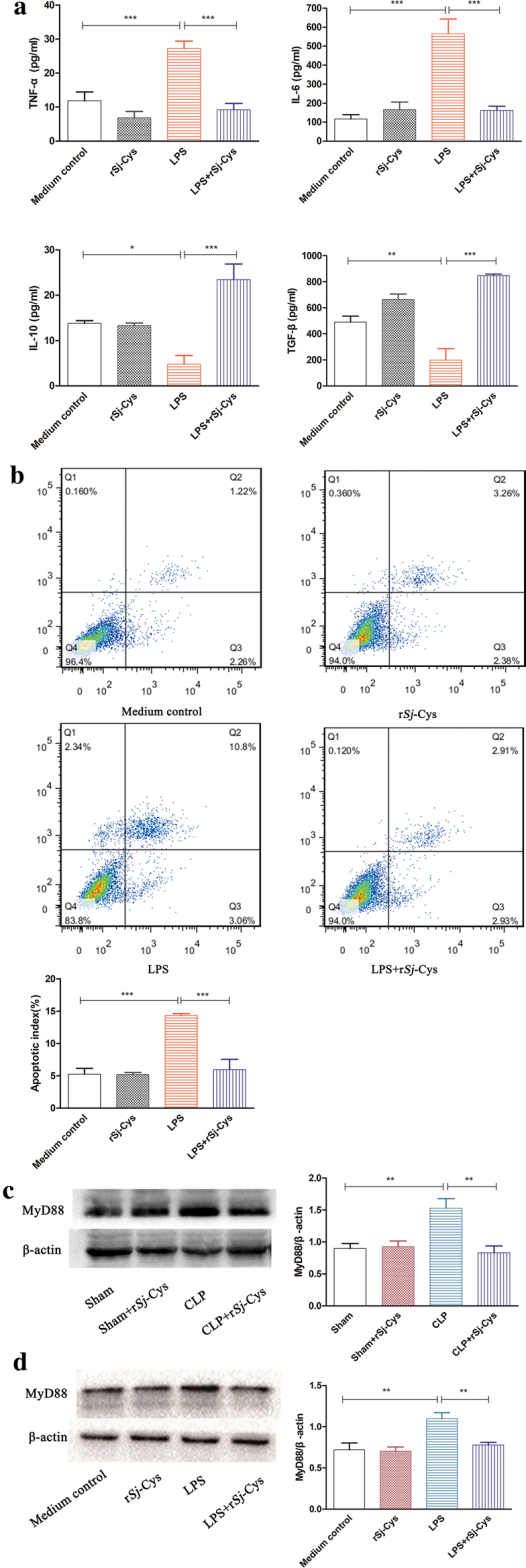
**a** Incubation with r*Sj*-Cys inhibited the pro-inflammatory cytokines TNF-α and IL-6 and stimulated regulatory cytokines IL-10 and TGF-β released by LPS-induced H9C2 cells. The levels of these cytokines in the supernatant were measured by ELISA 24 h after incubation. The results are shown as the mean ± SE for each group (*n* = 3 per group). **b** r*Sj*-Cys reduced LPS-induced cardiomyocyte apoptosis measured by flow cytometry. Representative flow cytometry images showed the reduced cardiomyocyte apoptosis in r*Sj*-Cys + LPS co-incubated H9C2 cells. The normal H9C2 cells in blank medium or medium with r*Sj*-Cys were used as controls. Data are expressed as mean ± SE from three independent experiments) (*n* = 3 per group). r*Sj*-Cys treatment suppressed the expression of MyD88 in the myocardial tissues of mice with CLP-induced sepsis (**c**) (*n* = 6 mice per group) and in LPS-incubated H9C2 cells (**d**) (*n* = 3 per group) measured by western blot. β-actin was measured as a control. The density ratio of MyD88/β-actin is shown on the right. The results are shown as the density mean ± SE for each group. *P < 0.05, **P < 0.01, ***P < 0.001

LPS is thought to be the major component to cause cardiac dysfunction in sepsis by inducing the innate immune inflammatory response [35]. In the present study, we identified that LPS significantly induced H9C2 cardiomyocytes to release pro-inflammatory cytokines IL-6 and TNF-α (ANOVA: *F*_(3, 11)_ = 20.78, *P *< 0.0004 and *F*_(3, 11)_ = 18.53, *P *< < 0.0006, respectively), but inhibited the release of regulatory cytokines TGF-β and IL-10 (ANOVA: *F*_(3, 11)_ = 25.67, *P *< 0.0002 and *F*_(3, 11)_ =14.41, *P *< 0.0014, respectively) compared with cells treated with PBS (Fig. 4a). After being treated with r*Sj*-Cys, the LPS-induced IL-6 and TNF-α were reduced to the level of cells unstimulated by LPS (ANOVA: *F*_(3, 11)_ = 20.78, *P *< 0.0004 and *F*_(3, 11)_ = 18.53, *P *< 0.0006, respectively), and TGF-β and IL-10 were significantly boosted compared to cells without r*Sj*-Cys treatment (ANOVA: *F*_(3, 11)_ = 25.67, *P *< 0.0002 and *F*_(3, 11)_ = 14.41, *P *< 0.0014, respectively) (Fig. 4a). The r*Sj*-Cys alone had no significant effect on the innate immune response of normal cardiomyocytes.

### r*Sj-*Cys reduced LPS-induced cardiomyocyte apoptosis

To further determine whether r*Sj-*Cys reduced LPS-induced cardiomyocyte apoptosis, H9C2 cells were incubated with LPS alone or with r*Sj-*Cys. The flow cytometry results revealed that incubation with LPS induced apoptosis in 14.4% of H9C2 cells, whereas co-incubation with r*Sj-*Cys significantly reduced LPS-induced apoptosis to a level similar to cells without LPS (ANOVA: *F*_(3, 11)_ = 22.68, *P *< 0.0003) (Fig. 4b). There was no significant difference in the apoptotic rate between the r*Sj-*Cys-alone group and blank control group.

### r*Sj*-Cys suppressed the activation of MyD88 in LPS-stimulated H9C2 cells *in vitro* and CLP-induced cardiac tissues *in vivo*

MyD88 is a crucial molecule involved in the inflammatory TLR signaling pathway. To determine if MyD88 is involved in the therapeutic effect of r*Sj*-Cys on sepsis-induced inflammation and damage of cardiomyocytes, we detected the expression of MyD88 in heart tissue of mice with CLP-induced sepsis treated with r*Sj-*Cys *in vivo*, and in LPS-stimulated H9C2 cells co-incubated with r*Sj*-Cys *in vitro*. The elevated level of MyD88 was observed in cardiac tissue 12 h after CLP surgery compared with the sham surgery control (ANOVA: *F*_(3, 11)_ = 8.823, *P *< 0.0064) (Fig. 4c). Treatment with r*Sj-*Cys significantly reduced the expression of MyD88 in cardiac tissue of sepsis mice compared with mice without treatment (ANOVA: *F*_(3, 11)_ = 8.823, *P *< 0.0064) (Fig. 4c). There was no effect of r*Sj*-Cys on the expression of MyD88 in normal mice (sham control). Meanwhile, the expression of MyD88 was also increased in cardiomyocytes incubated with LPS for 24 h. Co-incubation with r*Sj-*Cys significantly suppressed the expression of MyD88 in LPS-stimulated cardiomyocytes (ANOVA: *F*_(3, 11)_ = 8.550, *P *< 0.0071) (Fig. 4d).

## Discussion

Myocardial dysfunction is a fatal complication of patients with sepsis [18]. Studies have found that 50% of patients with sepsis have systolic and diastolic dysfunction in the heart left and right ventricle, possibly caused by endotoxin-induced myocardial injury [36] which has been confirmed both in animal and clinical observations [37]. In this study, we also confirm that CLP-induced sepsis caused serious myocardial damage characterized by a significant reduction of left ventricular systolic and diastolic functions in mice, which closely mimicked the pathological features of myocardial infarction observed in clinical patients [38]. We further confirm that LPS released by Gram-negative bacteria could cause apoptosis of H9C2 cardiomyocytes when co-incubated *in vitro*.

Cysteine proteases have been regarded as key molecules in regulating inflammation, cell apoptosis, cancer progression, protein degradation and antigen presentation [39–43]. Since cysteine proteases are largely involved in the inflammation and immune responses, their inhibitor, cystatin, could be a potential modulator for an immunological reaction. Actually, the cystatins secreted by several helminths have been proven to play important roles in modulating host immune responses [11, 21]. Previous studies demonstrated that *S. japonicum* cystatin (*Sj*-Cys) contained conserved domains of type II family cystatins with inhibitory activity on bovine cathepsin B [23]. *Sj*-Cys also inhibited LPS-stimulated macrophages to release nitric oxide, TNF-α and IL-6 cytokines and induced M2 macrophage polarization [23]. Treatment with r*Sj*-Cys significantly reduced TNBS-induced experimental colitis in mice through upregulation of Treg cells and related cytokines IL-10 and TGF-β, and downregulation of pro-inflammatory cytokines TNF-α and IL-6 in the colon tissues of mice [44].

In an effort to reduce sepsis-induced cardiomyopathy, the life-threatening complication and consequence of systemic infection, we established the mouse model of CLP-induced sepsis and sepsis-induced cardiomyopathy. We demonstrated that 12 h after CLP, the ventricular systolic and diastolic functions of affected mouse heart were seriously impaired associated with myocardial structural damage and inflammatory cell infiltration. Physiological changes in cardiac dysfunction include ventricular dilatation, decreased ejection fraction, systemic or regional left ventricular wall dysfunction and systolic and diastolic dysfunction [45]. EF%, FS% and the E/A ratio of the left ventricle (LV) are important indicators reflecting cardiac function [46, 47]. Echocardiographic results demonstrated that the CLP-induced sepsis resulted in a significant decrease in left ventricular EF, FS and E/A ratio, indicating the serious damage on heart systolic and diastolic functions. In addition, the levels of cTnI, NT-proBNP and Mb in sera and the MPO level in heart tissue are the important biochemical markers of cardiac damage and injury in early septic shock [32, 33, 48, 49]. The CLP-induced sepsis model established in this study demonstrated significantly increased levels of cTnI, NT-proBNP and Mb in sera and a high level of MPO in heart tissue, indicating that the heart tissue and cells were seriously damaged as a result of the CLP-induced sepsis. The significantly increased inflammatory cell infiltration in heart tissue also demonstrated the serious inflammation occurring in the heart.

After being treated with r*Sj*-Cys, the sepsis-induced heart malfunction has been significantly improved, showing a recovered left ventricular EF, FS and E/A ratio. The inflammation of heart tissue was also significantly reduced, as illustrated by the significantly decreased infiltration of inflammatory cells in cardiac tissues and fiber swelling. The levels of Mb, cTnI and NT-proBNP in sera were significantly reduced upon the treatment, indicating that r*Sj-*Cys attenuated endotoxin-induced myocardial damage and dysfunction. The MPO level was also reduced in heart tissue. Neutrophils play a significant role in the development of inflammation [50], and the activated neutrophils and monocytes are the main sources of MPO [51]. MPO is released into the blood as an inflammatory mediator, and promotes the activation of neutrophils, leading to further increased MPO and inflammation. Studies have also shown that neutrophil recruitment mediated myocardial injury and cardiac dysfunction induced by ischemia-reperfusion [52]. After treatment with r*Sj*-Cys, the levels of these biochemical markers of cardiac injury were significantly reduced, which was associated with the reduction of inflammatory cell infiltration in the heart and the proinflammatory cytokines (IL-6 and TNF-α) in sepsis mice.

Pro-inflammatory cytokines IL-6 and TNF-α play essential roles in the onset and progression of sepsis [53], and their over-expression was seen as an early signal suppressing myocardial contraction and the major cause of progressive systolic dysfunction [36, 54]. As a triggering factor of inflammation, TNF-α is also recognized as a main mediator of septic shock, and involved in the induction of IL-1 production, the latter induces the secretion of secondary inflammatory factors such as IL-6, resulting in an inflammatory cascade [55–57]. Further evidence has suggested that overexpressed IL-6 and TNF-α by systematic immune responses might support a vital role in the development of myocardial malfunction in sepsis [58]. At the same time, the septic cardiomyocytes were able to produce TNF-α and IL-6 themselves, indirectly leading to deteriorative damage to myocardial tissues [3]. The present study showed that treatment with r*Sj*-Cys significantly reduced the level of TNF-α and IL-6 in sera (systematic) and their mRNAs in cardiac homogenate (local), suggesting the regulatory effects of r*Sj*-Cys on local (cardiac) and systematic (sepsis) immune system.

Further evidence has shown that immunomodulatory functions of helminth infection or helminth-derived products are mediated by stimulating the host regulatory T cell (Treg) response [16, 17, 20]. Tregs are key factors in the induction of immune tolerance [59], mainly through the secretion of IL-10 and TGF-β to exert regulatory influence on the immune system [16]. IL-10 played a counter-regulatory effect in the inflammatory response and was an endogenous inhibitor of inflammatory cytokine production [60]. The levels of IL-10 and TGF-β were dramatically increased upon the treatment of r*Sj*-Cys in this study, indicating that r*Sj-*Cys acted as an inhibitory immunomodulator in the case of excessive inflammation infection possibly through stimulating Treg and Treg cell-secreted IL-10 and TGF-β. The r*Sj*-Cys itself had little effect on the cytokines change compared to the control mice without r*Sj*-Cys treatment, suggesting that r*Sj-*Cys mainly plays a immunomodulatory role when inflammation is activated. Incubation of r*Sj*-Cys with H9C2 cardiomyocytes *in vitro* inhibited LPS-induced heart cell apoptosis and induced similar cytokine changes in the culture supernatant to that in septic cardiomyopathy *in vivo*, further indicating that LPS-induced hyper-inflammatory responses resulted in cardiomyocyte apoptosis, and treatment with r*Sj*-Cys could inhibit LPS-triggered excessive inflammation partially through directly inhibiting the inflammatory cytokine IL-6 and TNF-α and stimulating the regulatory cytokines IL-10 and TGF-β in LPS-shocked cardiomyocytes. The reduced inflammatory cytokines may relatively contribute to the decline in myocardial apoptosis.

Another interesting finding in this study is that heart tissue from r*Sj*-Cys-treated sepsis-mice expressed more Arg-1 and less iNOS compared to the mice without r*Sj*-Cys treatment. Arg-1 is the biomarker for M2 alternatively activated macrophages that release anti-inflammatory cytokines to suppress immune responses and restore tissue homeostasis whereas iNOS is the biomarker of M1 classically activated macrophages responsible for the pro-inflammatory response. The shifting of pro-inflammatory M1 macrophages to anti-inflammatory M2 macrophages in the heart tissue of sepsis mice after being treated with r*Sj*-Cys may provide another mechanism involved in the reduced inflammation and heart damage caused by sepsis. M2-type macrophages have been reported to attenuate experimental inflammation in dinitrobenzene sulfonic acid (DNBS)-induced colitis in mice [61, 62].

LPS associates with its receptor, the toll-like receptor 4 (TLR4), through the help of LPS-binding protein CD14, subsequently resulting in the production of inflammatory cytokines, such as TNF-α, IL-1β and IL-18, which might directly harm cardiac function [63]. Although the mechanism leading to sepsis-induced cardiac arrest remains controversial, there is increasing evidence supporting that TLR-mediated innate immunity and inflammatory responses play a key role in cardiac dysfunction caused by sepsis or septic shock [64–66]. Activation of the TLR4 signaling pathway may directly lead to myocardial cells’ dysfunction. The invaded bacteria or other external stimulus first trigger innate immunity and then induce TLR4 expression by upregulating the MyD88-mediated pathway and activating the transcription of nuclear factor-κB (NF-κB), resulting in the production of various inflammatory mediators, such as cytokines, chemokines and antimicrobial peptides [67]. The occurrence of sepsis is related to the TLR4/MyD88 signaling pathway, which activates the secretion of cytokines associated with cardiac dysfunction in adult mammalian heart [7, 68] and in mice [69]. To investigate whether r*Sj*-Cys-involved anti-inflammatory and anti-apoptosis effects through inhibiting the MyD88-dependent signaling pathway, the level of MyD88 in CLP-induced septic heart tissue and in LPS-stimulated H9C2 cells was measured. Our study found that the expression of MyD88 increased in CLP-induced myocardial tissue or LPS-stimulated H9C2 cells, and treatment with r*Sj-*Cys significantly reduced the expression of MyD88 in these cardiomycytes. It is under investigation whether treatment of r*Sj*-Cys reduces TLR-2 or TLR-4 activation that results in downregulation of MyD88.

Our results suggest the possible mechanism of r*Sj-*Cys involved in the alleviation of septic cardiomyopathy is that treatment of r*Sj-*Cys stimulates Tregs and/or cardiomyocytes to produce regulatory cytokines such as IL-10 and TGF-β, and promotes the differentiation of M1 to M2 macrophages in heart tissue, thereby suppressing the production of pro-inflammatory cytokines *via* inhibiting the MyD88 activation signal pathway as shown for other helminth-derived proteins [70–73].

## Conclusions

The present data show that r*Sj*-Cys strongly alleviated excessive inflammation and protected against sepsis-induced cardiac dysfunction. Therefore, r*Sj*-Cys could be considered as a potential therapeutic agent for the prevention and treatment of sepsis associated cardiac dysfunction.

## Supplementary information

**Additional file 1: Table S1.** Primer sequences used for qRT-PCR analysis.

## Data Availability

The datasets supporting the findings of this article are included within the article and its additional file.

## Abbreviations

CLP: cecal ligation and puncture
LPS: lipopolysaccharides
Mb: myoglobin
cTnI: cardiac troponin I
NT-proBNP: N-terminal pro-Brain Natriuretic peptide
MPO: myeloperoxidase
MyD88: myeloid differentiation factor 88
EF: ejection fraction
FS: fractional shortening
E wave: peak early-diastolic transmitral velocities
A wave: peak late-diastolic transmitral velocities
LV: left ventricle
ELISA: enzyme-linked immunosorbent assay
TNF-α: tumor necrosis factor alpha
IL-6: interleukin 6
IL-10: interleukin 10
TGF-β: transforming growth factor-β
IL-1β: interleukin 1β
DCs: dendritic cells
Tregs: regulatory T cells
DNBS: dinitrobenzene sulfonic acid
DSS: dextran sulfate sodium
TLR4: toll-like receptor 4
NF-κB: nuclear factor-κB
SE: standard error of the mean
SPF: specific pathogen free
SDS-PAGE: sodium dodecyl sulfate polyacrylamide gel electrophoresis
PVDF: polyvinylidene fluoride
