# Supplementary material for: Therapeutic efficacy of Schistosoma japonicum cystatin on sepsis-induced cardiomyopathy in a mouse model
Source: Parasit Vectors. 2020 May 18;13:260. doi: 10.1186/s13071-020-04104-3 (PMC7236195; doi:10.1186/s13071-020-04104-3)
Supplement: Supplementary file 1 — Additional file 1: Table S1. Primer sequences used for qRT-PCR analysis. [file 13071_2020_4104_MOESM1_ESM.docx]

**Additional File 1: Table S1 Primer sequences used for qRT-PCR analysis.**

| **Gene** | **Forward primer** | **Reverse primer** | **References** |
| --- | --- | --- | --- |
| TNF-α | 5′- AACCTCCTCTCTGCCGTCAA -3′ | 5′-AAAGTAGACCTGCCCGGACTC -3′ |  |
| IL-6 | 5′-TGGAGTCACAGAAGGAGTGGCTAA -3′ | 5′-TCTGACCACAGTGAGGAATGTCCA -3′ |  |
| TGF-β | 5′-CTACAATGAGCTGCGTGTG -3′ | 5′-TGGGGTGTTGAAGGTCTC -3′ | Chen ZB et al. [29] |
| IL-10 | 5′-CCAAGCCTTATCGGAAATGA -3′ | 5′-TTTTCACAGGGGAGAAATCG -3′ | Chen ZB et al. [29] |
| iNOS | 5′-CAAGCACCTTGGAAGAGGAG -3′ | 5′-AAGGCCAAACACAGCATACC -3′ | Jia L et al. [30] |
| Arg-1 | 5′-CTCCAAGCCAAAGTCCTTAGAG -3′ | 5′-AGGAGCTGTCATTAGGGACATC -3′ | Cheng Y et al. [31] |
| GAPDH | 5′-GGTTGTCTCCTGCGACTTCA -3′ | 5′-TGGTCCAGGGTTTCTTACTCC -3′ |  |
